# Optical magnification has no benefits on the detection of occlusal caries lesions in permanent molars using different visual scoring systems: An in vitro study

**DOI:** 10.4317/jced.56445

**Published:** 2020-05-01

**Authors:** Sabrina Wilde, Priscila-Hernández Campos, Ana-Paula-Marçal Marcondes, Cacio Moura-Netto, Tatiane-Fernandes Novaes, Adrian Lussi, Michele-Baffi Diniz

**Affiliations:** 1 School of Dentistry, Federal University of Rio Grande do Sul, Porto Alegre, RS, Brazil; 2 Post-graduate Program in Dentistry, Cruzeiro do Sul University - São Paulo, SP, Brazil; 3 Faculty of Dental Medicine, Center for Interdisciplinary Research in Health (CIIS), Universidade Católica Portuguesa, Viseu, Portugal; 4 Center for Dental Medicine, Medical Center, University of Freiburg - Freiburg, Germany

## Abstract

**Background:**

Some studies have addressed the influence of optical magnification on the detection of caries lesions using a visual scoring system. However, there is a lack of research related to the use of the CAST and ADA-CCS visual scoring systems. In addition, the reliability and accuracy of ADA-CCS index in permanent teeth were not studied yet. So, the aim of this study was to evaluate, in vitro, the influence of different levels of optical magnification on the detection of occlusal caries lesions in permanent molars using three visual scoring systems.

**Material and Methods:**

One occlusal site per tooth was analyzed in 120 extracted permanent molars. Two trained examiners inspected the teeth using ICDAS (International Caries Detection and Assessment System), CAST (Caries Assessment Spectrum and Treatment), and ADA-CCS (American Dental Association-Caries Classification System) visual criteria, twice with each scoring system, with a one-week interval between examinations. The study was conducted in three phases: (A) without optical magnification, (B) using a binocular lens (3.5× magnification), and (C) using an operating microscope (16× magnification). Then, the teeth were sectioned longitudinally through the center of the selected site and the section with the more severe lesion was histological evaluated considering the D1 (lesions in enamel and dentin) and D3 (dentin lesions) thresholds.

**Results:**

Kappa values for intra- and inter-examiner reproducibility were good to excellent for all systems. At the D1 threshold, sensitivity, accuracy, and area under the ROC curve were high for ICDAS and CAST in all phases. However, this was not the case for the ADA-CCS in phase C (**<0.05). At the D3 diagnostic threshold, there was no significant difference between the visual scoring systems during the study phases (**>0.05).

**Conclusions:**

The magnification does not improve the accuracy of the visual scoring systems in the detection of occlusal caries lesions in permanent molars.

** Key words:**Dental caries, caries detection, permanent teeth, visual examination, magnification.

## Introduction

Visual examination is the most commonly used method for detecting caries lesions. It is based on subjective criteria including color, translucency, and hardness of the dental structure. This method has high specificity but relatively low sensitivity. However, the use of well-established visual criteria allows for increased sensitivity and accuracy (1). ICDAS (International Caries Detection and Assessment System), CAST (Caries Assessment Spectrum and Treatment) and ADA-CCS (American Dental Association-Caries Classification System) are visual scoring systems that have been used in cariology studies (2-6).

The ICDAS system evaluates six stages of the carious process, from initial changes visible in enamel to extensive cavitation with dentin involvement (2,7). In vitro and *in vivo* studies show that the use of ICDAS provides good reproducibility and accuracy in the detection of occlusal caries lesions in permanent teeth (1,8-11). The CAST system includes eleven hierarchically ordered scores; it combines elements of other systems and is being proposed for use in epidemiological studies. It has shown promising results, with a more precise analysis of different stages of caries lesions when compared to DMF-T/dmf-t (5,12,13). Finally, the ADA-CCS system was developed to provide a classification of the entire range of caries (healthy, or exhibiting initial, moderate and advanced caries lesions) and its impact on patient care for use in daily practice (4,14). Although this system presents some similarities with the ICDAS criteria, the performance of the ADA-CCS has not yet been investigated. So, this is the first study evaluating the reliability and accuracy of this system in permanent teeth.

It is well known that the progression of initial caries lesions can be arrested with preventive and non-invasive treatments. However, the detection of non-cavitated lesions and micro-cavities in enamel is a difficult task, and any inconsistency in diagnosis will inevitably lead to treatment variability (15). In addition, mistakes with respect to dentin caries detection could lead to unnecessary operative treatments. So, to help to clarify these challenges, clinicians have turned to the use of magnification to aid in caries diagnosis (16-21) and restorative treatment decision-making (18,22,23) because it provides a clear visualization of the carious site (16). This study examines the use of magnification in the caries diagnosis process and is the first to evaluate the influence of magnification on the use of CAST and ADA-CCS systems.

The aim of this study was to evaluate *in vitro* the influence of different levels of optical magnification (binocular lens, 3.5×; operating microscope, 16×) compared to no magnification on the detection of occlusal caries lesions in permanent teeth using three visual scoring systems (ICDAS, CAST and ADA-CCS). The hypothesis of the study was that optical magnification does not improve the detection of caries lesions on the occlusal surface.

## Material and Methods

This study was approved by the local Research Ethics Committee (Protocol 042/2015). We used 120 permanent human third molars, which were extracted for reasons unrelated to this study. The teeth were donated by patients of the Odontology Clinic in São Paulo, Brazil. Prior to extraction, the patients provided an informed consent about the use of their teeth for research purposes.

We included teeth with macroscopically healthy occlusal surfaces (n=21) or with suspected carious lesions (n=67 with non cavitated lesions and n=32 with cavitated lesions). Teeth with occlusal restorations or with pit and fissure sealants, hypoplasia, fluorosis, crown destruction or extensive caries lesions on other surfaces were excluded from the study.

The teeth were cleaned and storaged, individually, in 0.1% thymol solution and refrigeration at 4 °C to inhibit bacterial growth. So, the occlusal surfaces were photographed using a digital camera (Canon EOS, Japan). An independent examiner, who did not participate in the evaluation, selected one site on the occlusal surface of each tooth. The site more susceptible to caries or presenting the severest condition was selected and identified on black-and-white printouts of each tooth.

Two examiners (S.W. and P.H.C.) who were trained and familiar with the visual criteria performed the examinations. As the examiners were not familiar with the use of magnification, the manufacturer’s instructions were also presented and discussed in the training sessions to allow for correct adjustment of the settings (resolution, working distance, field of view, depth of field and interpupillary distance) of the binocular lens and operating microscope.

The study was conducted in three phases: (A) examination under halogen light from the reflector (Model Persus L, intensity 8,000 to 25,000 LUX, Gnatus, Ribeirão Preto-SP, Brazil), without optical magnification; (B) examination with a binocular lens (420 mm lens) of 3.5× magnification and under an LED headlight (Denshine, China); and (C) examination with a wall-mounted 16× magnification operating microscope (200 mm objective - ALL 02 Operating Microscope, Alliance, São Carlos-SP, Brazil) and maximum light intensity (2 halogen bulbs of 15V/150W).

During each stage of the study, the examiners each evaluated the teeth twice using each visual scoring system, with one-week intervals between examinations. The teeth were placed on a portion of modeling clay with a cotton roll placed over the crown to maintain natural moisture. There was no time limit on the examination, and the sequence of the examination was reversed randomly at each stage to avoid bias. For the visual evaluations without optical magnification (phase A), it was considered a working distance of approximately 300 mm. During the examination with a binocular lens (phase B), a working distance of 350 mm was established; with the operating microscope (phase C), a working distance of 250 mm was used. Glasses were used by the examiners if necessary (24).

When using the ICDAS criteria, examinations were performed with a reflector light, a triple syringe, and a WHO ball-end probe. Each surface was classified according to the scoring system proposed by Ismail *et al.* (7) as: 0) sound tooth surface; 1) first visual change in enamel; 2) distinct visual change in enamel observed when wet; 3) localized enamel breakdown; 4) underlying dark shadow from dentin; 5) distinct cavity with visible dentin, involving less than half of a tooth surface; and 6) extensive distinct cavity, involving more than half of the tooth. When using the CAST criteria, the occlusal surfaces were not dried with compressed air; excess water was removed with gauze or a cotton roll, and the WHO ball-end probe was used. This was followed by an examination under a reflector light. Scores of 0, 3, 4, 5, and 6 were used in the *in vitro* evaluation, as described by de Souza *et al.* (3): 0) no visible evidence of a distinct caries lesion is present; 3) a distinct visual change in enamel only; 4) internal caries-related discoloration in dentin; 5) distinct cavitation into dentin, but the pulp chamber is intact; and 6) involvement of the pulp chamber. For examinations using ADA-CCS criteria, a reflector light, a triple syringe, and a WHO ball-end probe were used, as described by Young *et al*. (14): 0) no clinically detectable lesion; 1) earliest clinically detectable lesion compatible with mild demineralization; 2) visible signs of enamel breakdown or signs the dentin is moderately demineralized; and 3) enamel is fully cavitated and dentin is exposed.

After evaluation, the teeth were sectioned longitudinally through the center of the selected site with a precision cutting machine (Isomet 1000, Buehler, Lake Bluff, IL, USA), resulting in two sections corresponding to the test site that were sanded longitudinally in a rotary electric polishing machine (Ecomet, Buehler, Lake Bluff, IL, USA).

Histological analyses were performed with a stereomicroscopic magnifying glass according to the extent of the lesion described by Downer (25): 0) no enamel demineralization; 1) enamel demineralization limited to the outer 50% of the enamel layer; 2) demineralization involving the inner 50% of the enamel; 3) demineralization involving the outer 50% of the dentin; and 4) demineralization involving the inner 50% of the dentin. Dental sections were individually analyzed by two experienced observers who were not involved in the clinical examination (A.P.M.M. and M.B.D.). In cases of disagreement, additional evaluations were performed until a consensus.

Data were analyzed using the MedCalc 15.2.2 (Mariakerke, Belgium), with a significance level set at 5%. Intra- and inter-examiner reproducibility was calculated for the three visual scoring systems at each study stage. The weighted Cohen Kappa coefficient and 95% confidence interval were calculated for ICDAS, CAST, and ADA-CCS. Kappa values above 0.75 were considered excellent agreement, while values between 0.40 and 0.75 were considered good agreement (8).

Sensitivity, specificity, accuracy, and area under the ROC curve (Az) were calculated at the diagnostic D1 threshold (lesions in enamel and dentin) and at D3 (dentin lesions) for the three visual scoring systems in each study phase. For the D1 threshold, ICDAS scores 1-6, CAST scores 3-6, and ADA-CCS scores 1-3 scores were considered. For the D3 threshold, ICDAS scores 3-6, CAST scores 4-6, and ADA-CCS scores 2-3 were considered. The McNemar test was used to compare the results for each study phase. A non-parametric test compared differences between Az values. Crossed 2×2 Tables were constructed to compare the three visual scoring systems and a histological analysis. The Spearman correlation coefficient evaluated the correlation between the methods and a histological analysis. A coefficient of ≥ 0.70 indicated a strong correlation between variables (8).

## Results

We evaluated 120 teeth and the histological results showed that 7 sites (5.8%) had a score of 0, 28 (23.3%) had a score of 1, 43 (35.9%) had a score of 2, 18 (15.0%) had a score of 3, and 24 (20.0%) had a score of 4.

We observed good (0.617) to excellent agreement (0.916) among the three criteria and among the three evaluation phases, with good intra- and inter-examiner reproducibility. In general, we observed a slight decrease in weighted Kappa values for intra- and inter-examiner reliability with increasing magnification.

Table 1 shows the performance values for each visual scoring system with respect to the three study phases for the diagnostic thresholds D1 and D3, respectively. At the D1 threshold, in general, sensitivity, accuracy, and area under the ROC curve (Az) were high for the ICDAS, CAST and ADA-CCS systems in all study phases. In addition, the specificity values were lower than the sensitivity values for all visual criteria, in all phases of the study, being the lowest value found by ADA-CCS when using the operating microscope (0.39). At the D3 threshold, there was no statistically significant difference between the visual scoring systems during the three study phases (*p*>0.05). However, the CAST system presented higher specificity and lower sensitivity.

Table 1 shows the performance values for each visual scoring system with respect to the three study phases for the diagnostic thresholds D1 and D3, respectively. At the D1 threshold, in general, sensitivity, accuracy, and area under the ROC curve (Az) were high for the ICDAS, CAST and ADA-CCS systems in all study phases. In addition, the specificity values were lower than the sensitivity values for all visual criteria, in all phases of the study, being the lowest value found by ADA-CCS when using the operating microscope (0.39). At the D3 threshold, there was no statistically significant difference between the visual scoring systems during the three study phases (*p*>0.05). However, the CAST system presented higher specificity and lower sensitivity.

**Table 1:**
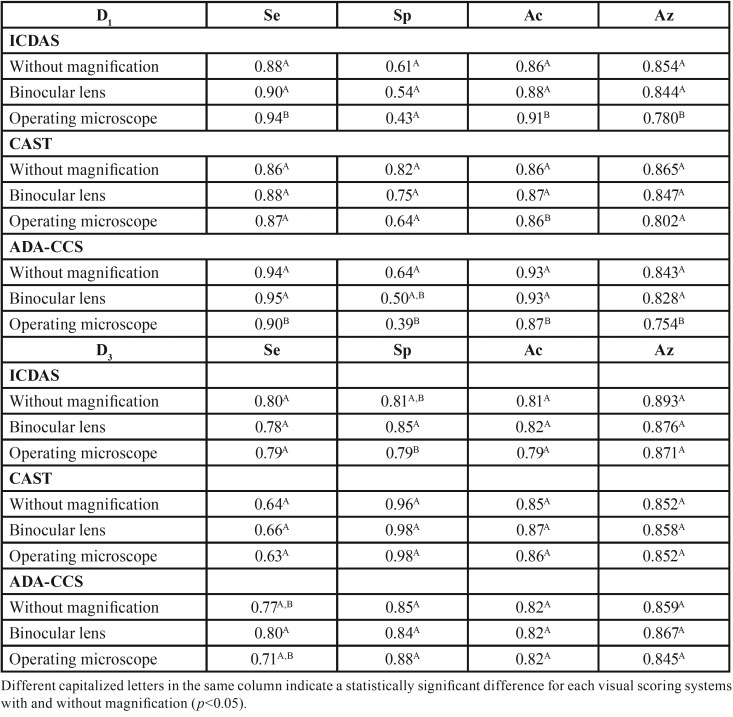
Sensitivity (Se), specificity (Sp), accuracy (Ac), and area under the ROC curve (Az) for the three visual scoring systems in each study phase for diagnostic thresholds D1 (lesions in enamel and dentin) and D3 (dentin lesions).

 Table 2,  Table 3 and  Table 4 show the correlation between the visual scoring systems (without magnification, with a binocular lens, and with an operating microscope, respectively) and the histological analysis. We observed that for the all three different visual scoring systems, the higher the magnification used, the lower the number of teeth was classified as healthy. Consequently, with increasing magnification, many enamel lesions were identified on surfaces that were considered healthy in the histological examination.

**Table 2:**
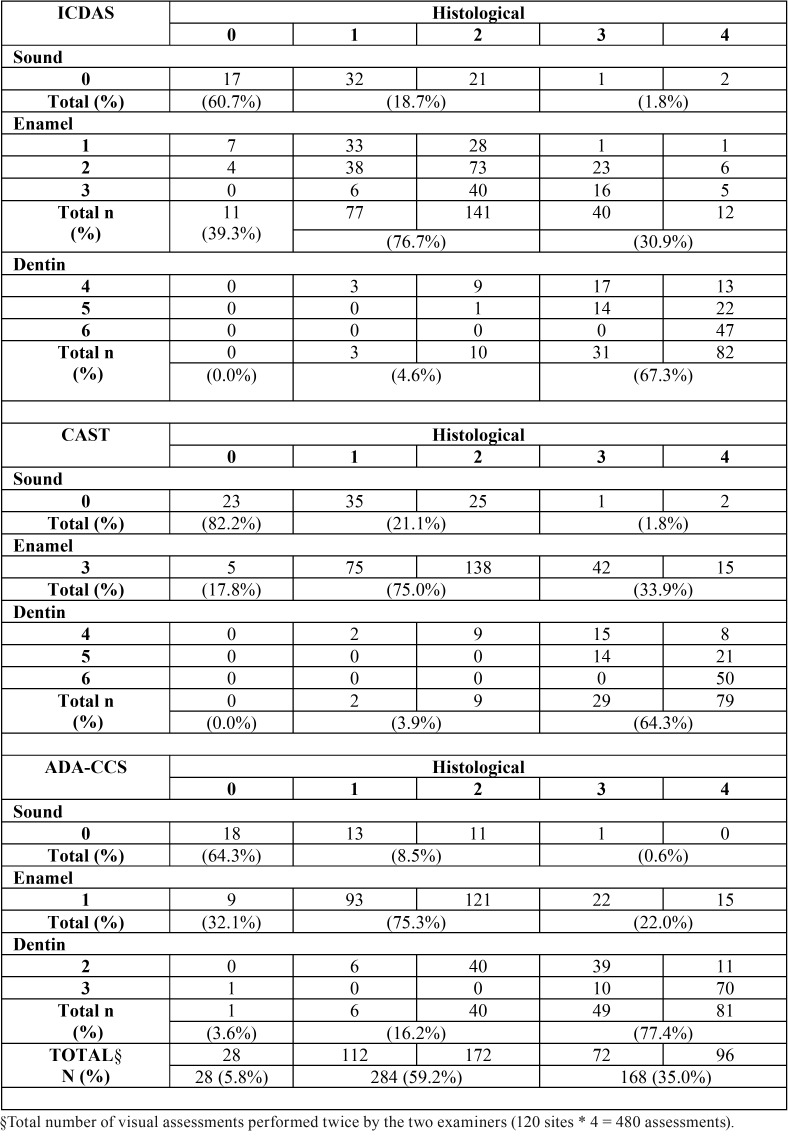
Cross tabulation between the three visual scoring systems and the histological analysis for the study phase A (without magnification).

**Table 3:**
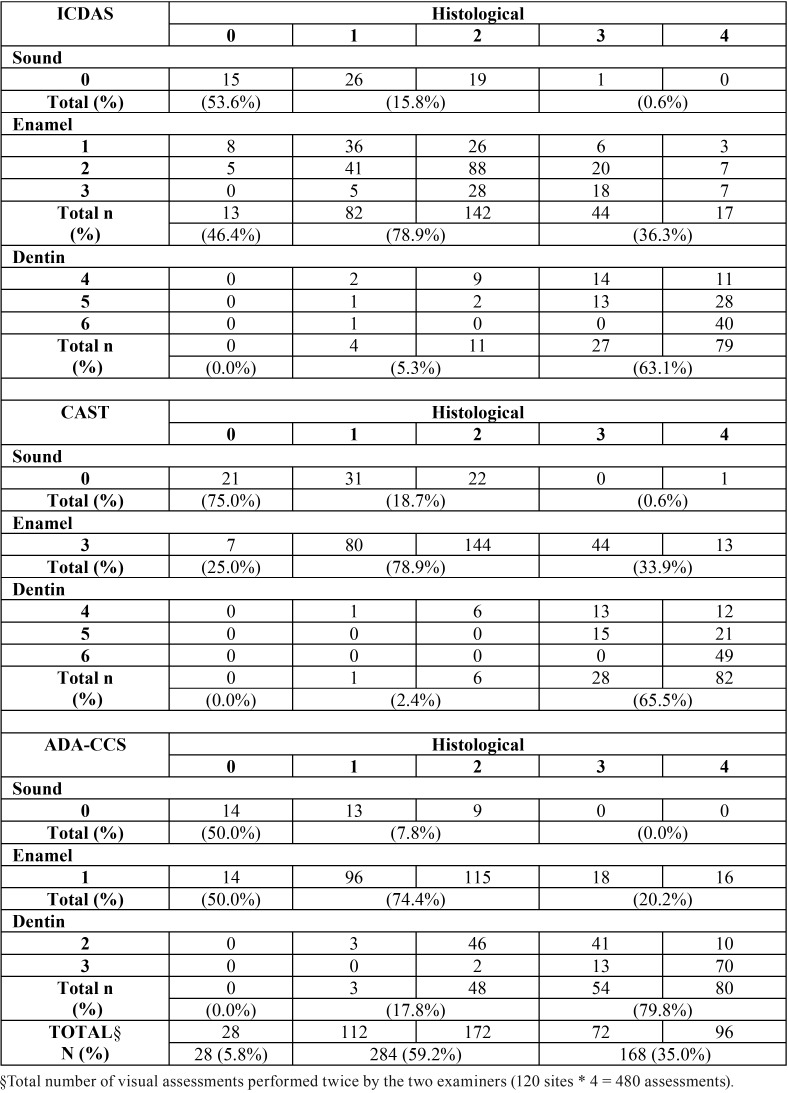
Cross tabulation between the three visual scoring systems and the histological analysis for the study phase B (with a binocular lens).

**Table 4:**
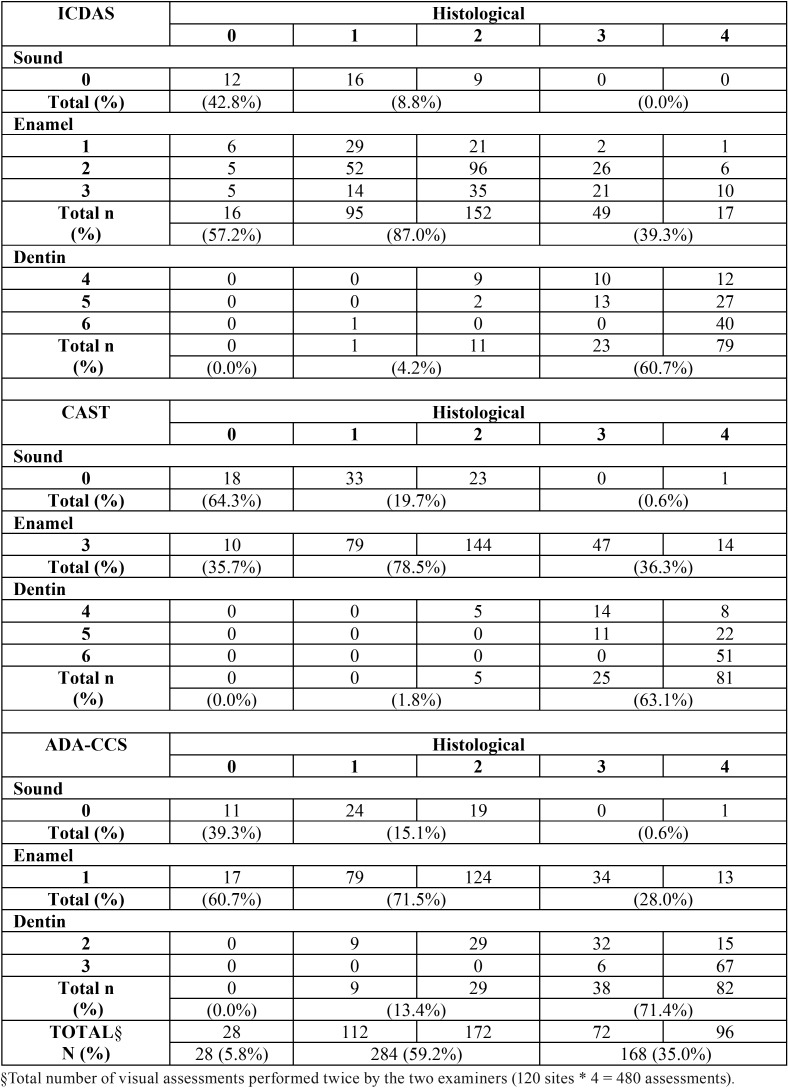
Cross tabulation between the three visual scoring systems and the histological analysis for the study phase C (with an operating microscope).

Regarding the identification of enamel lesions, without magnification (ICDAS scores 1 to 3, CAST score 3, and ADA-CCS score 1) the presence of these lesions was confirmed by the histological examination (scores 1 and 2), in 75% to 76.7% of the cases. On the other hand, the identification of enamel lesions with a binocular lens was confirmed by the histological examination in 74.4% to 78.9% of the cases. With the operating microscope, the presence of these lesions was confirmed in 71.5% to 87.0% of the cases. With regard to the dentin lesions without magnification (ICDAS and CAST scores 4 to 6 or ADA-CCS scores 2 and 3) the presence of these lesions was confirmed by the histological examination (scores 3 and 4) in 64.3% to 77.4% of cases. Similarly, the identification of dentin lesions with a binocular lens was confirmed in 63.1% to 79.8% of the cases. With the operating microscope, the dentin lesions were confirmed in 60.7% to 71.4% of the cases.

So, a marginal effect of magnification was observed only in the case of shaded lesions (score 4 according to ICDAS and CAST), when the higher the magnification, the lower the number of lesions confirmed as dentin lesions by the histological examination (scores 3 and 4). In the identification of advanced dentin lesions, without magnification, only one lesion classified as score 5 using the ICDAS criteria was restricted to enamel on histological evaluation (score 2). One surface classified as healthy using all visual systems was validated as dentin lesion on histological evaluation (score 3) and 2 surfaces classified as healthy using ICDAS and the CAST criteria were validated as advanced dentin lesions on histological evaluation (score 4). With the binocular lens 4 cases and with the operating microscope 3 cases judged as advanced dentin lesions using ICDAS (scores 5 and 6) were validated as enamel lesions on histological evaluation (scores 1 and 2). The advanced dentin lesions classified by the CAST criteria (scores 5 and 6), with or without the use of magnification, were truly validated as dentin lesions on histological evaluation (scores 3 and 4).

With the ICDAS, a higher correlation was found without magnification (0.725) and the lower value was related to operating microscope (0.680). Regarding the CAST, the correlation values were similar (0.701 without magnification; 0.714 for Binocular Lens and 0.709 for microscope). The ADA-CCS was lower correlated to histological founds when was used the operating microscope (0.654). With the binocular lens and without magnification, the correlations were 0.712 and 0.693, respectively.

## Discussion

To date, many authors have evaluated the effect of use of magnification on the treatment decision process (18,22,23), whereas the present study evaluated its effect on the diagnostic process. This is particularly relevant because the criteria evaluated in the present study assess caries lesions based on their different stages of development (17,19,20,26). In addition, there is not enough research investigating the effect of magnification on the use of CAST and ADA-CCS visual scoring systems for detecting caries lesions on permanent teeth. In the present study, we confirmed the hypothesis that the use of magnification does not improve the detection of occlusal caries lesions using ICDAS, CAST and ADA-CCS visual scoring systems.

In general, we found good agreement between different visual scoring systems, with or without optical magnification. Indeed, the practice and training required for the precise application of validated criteria to assess and classify different stages of caries lesions improves the reproducibility and overall performance of the method (1). There have been reports on the reliable application of the ICDAS (2,7) and CAST criteria (13). However, Mitropoulous *et al*. (20) reported a reduction in inter-examiner reproducibility with the use of a bifocal lens (×2.8) in the detection of ICDAS score 1 lesions on the occlusal surfaces of permanent teeth. The authors attribute this to the possible presence of pigmentation and other non-carious defects (e.g., developmental), which may lead to an erroneous classification of early enamel lesions.

Regarding the lower Kappa values found in the assessment of inter-examiner reproducibility, intensive training sessions were performed to minimize this variability. Zafersoy-Akarslan *et al.* (19) suggest that this is a relatively common finding because of variability in experience of evaluators. In the present study, both examiners were enrolled in a post-graduate program in dentistry with more than 3 years of clinical experience in dental caries diagnosis. However, no examiner had experience in the use of optical magnification, which could be influenced the results. In addition, the caries detection relies on a subjective evaluation of each dental professional that may have a different interpretation of the dental surfaces’ characteristics and of the signs of the caries disease based on visual acuity, experience or education (11).

This *in vitro* study was the first to evaluate the use of ADA-CCS criteria in the detection of occlusal caries lesions in permanent teeth. For the detection of enamel lesions without magnification, we found high values of sensitivity, accuracy and Az, but low values of specificity. However, the use of the operating microscope decreased the accuracy of the ADA-CCS system, emphasizing that the magnification should not be indicated for caries detection using this criterion.

The use of magnification in the detection of caries lesions has inherent limitations, such as a reduction in the field and depth of vision (21,23). Erten *et al.* (17) found that the use of an operating microscope with a magnification of 16× increases the number of false-positive diagnoses. This is in agreement with the findings of our study as shown in Tables  Table 2 to  Table 4. The use of an operating microscope may have overestimated caries lesions, especially using the ICDAS visual criteria, with sound surfaces being assessed as having non-cavitated lesions (D1 threshold) with an increased level of magnification.

In addition to the correlation of visual scoring system-related data with histological findings, the limitation in identifying healthy surfaces resulted in an overestimation of enamel lesions for all the scoring systems (ICDAS scores 1 to 3, CAST score 3, and ADA-CCS score 1). Consequently, we believe that when histologically healthy surfaces are erroneously classified as enamel lesions, it leads to unnecessary therapeutic and control measures with additional costs. Any inconsistency in diagnosis inevitably leads to undesirable changes in treatment (15).

Although this overestimation was not significant with respect to the detection of dentin lesions, we observed that in the case of shaded lesions (code 4 according to ICDAS and CAST), the higher the magnification, the lower the number of lesions identified. The exclusion of extensively cavitated lesions in dentin during sample selection may also have influenced these results. Nevertheless, with the use of ICDAS and ADA-CCS criteria, we noticed that some cases judged as advanced dentin lesions were shown to be healthy or restricted to the enamel according to the histological examination; the number of such lesions increased with an increase in the level of magnification. In fact, the use of magnification for detecting occlusal caries lesions in permanent molars, especially under an operating microscope, increases the number of false-positive results and may consequently result in overtreatment (17). In addition, the similarities between ADA-CCS and ICDAS criteria could explain these results. The ADA-CCS and ICDAS used in the protocol of the ICCMS (International Caries Classification and Management System) (27), classify the clinical stage of the carious lesions in a more objective caries categories, being the visible signs of discontinuity in the enamel and the presence of signs of shading or the presence of dentin demineralization considered moderate stage caries.

The incidence of diagnostic mistakes can occur, especially with low specificity values as observed for all three scoring systems. Baelum (28) also reported that the increase in false-positive results due to the low specificity of the diagnostic method may lead to a substantial increase in the indication for invasive treatment. Accordingly, the best diagnostic method should result in patient-centered outcomes in terms of better oral health in the long run.

Although the correlation between visual scoring systems and histological analysis or even the values of sensitivity, accuracy and the values of the Spearman correlation coefficients have remained similar to some extent regardless of the use of magnification, studies that have used magnification have shown an increase in the accuracy of the visual examination for the detection of caries lesions (16,26) and for making treatment decisions (18,22), or similar results (20,23). On the other hand, the risk of false-positive diagnoses has also been highlighted (26). Such differences can be explained by the use of different methodologies, different sample selection criteria or heterogeneous magnification levels. With the use of indices and visual criteria, differences in interpretation may also occur because of variability in the visual perception of evaluators or differences in the source of illumination (24,26). In the present study, different lighting sources, specific to the application of each method, may also have interfered with the accuracy of different methods, thus leading to over or underestimation of caries lesions in the results.

In the future, clinical studies need to be performed to evaluate the effect of different levels of magnification (e.g., a binocular lens and an operating microscope) on the detection of occlusal caries lesions and consequent treatment decisions.

In conclusion, the use of magnification does not improve the visual detection of occlusal caries lesions in permanent teeth. As the higher the magnification used, higher diagnostic errors can be found.

## References

[1] Gimenez T, Piovesan C, Braga MM, Raggio DP, Deery C, Ricketts DN (2015). Visual inspection for caries detection: a systematic review and meta-analysis. J Dent Res.

[2] Ekstrand KR, Martignon S, Ricketts DJ, Qvist V (2007). Detection and activity assessment of primary coronal caries lesions: a methodologic study. Oper Dent.

[3] de Souza AL, van der Sanden WJM, Leal SC, Frencken JE (2012). Caries Assessment Spectrum and Treatment (CAST) index: face and content validation. Int Dent J.

[4] Fisher J, Glick M, FDI World Dental Federation Science Committee (2012). A new model for caries classification and management: the FDI World Dental Federation caries matrix. J Am Dent Assoc.

[5] Malik A, Shaukat MS, Qureshi A (2014). Prevalence of dental caries using novel caries assessment index - CAST. J Dow Univ Health Sci Karachi.

[6] Leal SC, Ribeiro APD, Frencken JE (2017). Caries Assessment Spectrum and Treatment (CAST): A novel epidemiological instrument. Caries Res.

[7] Ismail AI, Sohn W, Tellez M, Amaya A, Sen A, Hasson H (2007). The International Caries Detection and Assessment System (ICDAS): an integrated system for measuring dental caries. Community Dent Oral Epidemiol.

[8] Jablonski-Momeni A, Stachniss V, Ricketts DN, Heinzel-Gutenbrunner M, Pieper K (2008). Reproducibility and accuracy of the ICDAS-II for detection of occlusal caries in vitro. Caries Res.

[9] Rodrigues JA, Hug I, Diniz MB, Lussi A (2008). Performance of fluorescence methods, radiographic examination and ICDAS II on occlusal surfaces in vitro. Caries Res.

[10] Diniz MB, Boldieri T, Rodrigues JA, Santos-Pinto L, Lussi A, Cordeiro RC (2012). The performance of conventional and fluorescence-based methods for occlusal caries detection: an in vivo study with histologic validation. J Am Dent Assoc.

[11] Bottenberg P, Jacquet W, Behrens C, Stachniss V, Jablonski-Momeni A (2016). Comparison of occlusal caries detection using the ICDAS criteria on extracted teeth or their photographs. BMC Oral Health.

[12] Baginska J, Rodakowska E, Milewski R, Kierklo A (2014). Dental caries in primary and permanent molars in 7-8-year-old schoolchildren evaluated with Caries Assessment Spectrum and Treatment (CAST) index. BMC Oral Health.

[13] de Souza AL, Leal SC, Bronkhorst EM, Frencken JE (2014). Assessing caries status according to the CAST instrument and WHO criterion in epidemiological studies. BMC Oral Health.

[14] Young DA, Nový BB, Zeller GG, Hale R, Hart TC, Truelove EL (2015). The American Dental Association Classification System for clinical practice. J Am Dent Assoc.

[15] Al-Khatrash AA, Badran YM, Alomari QD (2001). Factors affecting the detection and treatment of occlusal caries using the International Caries Detection and Assessment System. Oper Dent.

[16] Forgie AH, Pine CM, Pitts NB (2002). The use of magnification on a preventive approach to caries detection. Quintessence Int.

[17] Erten H, Üçtasli MB, Akarslan ZZ, Uzun O, Baspinar E (2005). The Assessment of unaided visual examination, intraoral camera and operating microscope for the detection of occlusal caries lesions. Oper Dent.

[18] Akarslan ZZ, Erten H (2009). The use of a microscope for restorative treatment decision-making on occlusal surfaces. Oper Dent.

[19] Zafersoy-Akarslan Z, Erten H, Uzun O, Semiz M (2009). Reproducibility and agreement of clinical diagnosis of occlusal caries using unaided visual examination and operating microscope. J Can Dent Assoc.

[20] Mitropoulos P, Rahiotis C, Kakaboura A, Vougiouklakis G (2012). The impact of magnification on occlusal caries diagnosis with implementation of the ICDAS II criteria. Caries Res.

[21] Neuhaus KW, Jost F, Perrin P, Lussi A (2015). Impact of different magnification levels on visual caries detection with ICDAS. J Dent.

[22] Erten H, Uçtasli MB, Akarslan ZZ, Uzun O, Semiz M (2006). Restorative treatment decision making with unaided visual examination, intraoral camera and operating microscope. Oper Dent.

[23] Sisodia N, Manjunath MK (2014). Impact of low level magnification on incipient occlusal caries diagnosis and treatment decision making. J Clin Diag Res.

[24] Neuhaus KW, Jasarevic E, Lussi A (2015). Impact of different illumination conditions on visual caries detection with ICDAS. Caries Res.

[25] Downer MC (1975). Concurrent validity of an epidemiological diagnostic system for caries with the histological appearance of extracted teeth as validating criterion. Caries Res.

[26] Ari T, Ari N (2013). The performance of ICDAS-II using low-powered magnification with Light-Emitting Diode headlight and alternating current impedance spectroscopy device for detection of occlusal caries on primary molars. ISRN Dent.

[27] Ismail AI, Pitts NB, Tellez M, Authors of International Caries Classification and Management System ICCMS, Banerjee A, Deery C (2015). The International Caries Classification and Management System (ICCMS™) an example of a caries management pathway. BMC Oral Health.

[28] Baelum V (2010). What is an appropriate caries diagnosis?. Acta Odontol Scand.

